# The association of post-stroke changes in body mass index with activity of daily living and instrumental activity of daily living trajectories: A multi-cohort analysis

**DOI:** 10.1016/j.jnha.2026.100772

**Published:** 2026-01-12

**Authors:** Guillaume Chambinaud, Aurore Fayosse, Aline Dugravot, Benjamin Landré, Alexis Schnitzler, Archana Singh-Manoux, Séverine Sabia, Louis Jacob

**Affiliations:** a Université Paris Cité, Inserm U1153, CRESS, Epidemiology of Ageing and Neurodegenerative Diseases (EpiAgeing), Paris, France; b Assistance Publique – Hôpitaux de Paris (AP-HP), Université Paris Cité, Lariboisière-Fernand Widal Hospital, Department of Physical Medicine and Rehabilitation, Paris, France; c Faculty of Brain Sciences, University College London, United Kingdom; d Research and Development Unit, Parc Sanitari Sant Joan de Déu, CIBERSAM, ISCIII, Dr. Antoni Pujadas, 42, Sant Boi de Llobregat, Barcelona, Spain

**Keywords:** Activities of daily living, Body mass index, Epidemiology, Instrumental activities of daily living, Stroke

## Abstract

**Objectives:**

The determinants of functional limitation trajectories after stroke remain scarce. This study aimed to investigate the association of early body mass index (BMI) changes with trajectories of activities of daily living (ADLs) and instrumental activities of daily living (IADLs) following stroke.

**Design:**

Three cohorts from Europe and the United States.

**Setting:**

Community.

**Participants:**

Stroke survivors.

**Measurements:**

BMI changes were assessed 1–4 years after self-reported stroke and categorized as decreased (≤−5% initial BMI), increased (≥5% initial BMI), and stable. An alternate cut-point of 2% was also used. Functional limitations were measured as the number of ADL and IADL limitations, which were repeatedly measured for up to 24 years after stroke. Associations were evaluated using segmented linear mixed-effects models after adjusting for demographic, behavioral, and medical factors.

**Results:**

The study population comprised 2544 adults with stroke (mean [standard deviation] age 70.0 [10.9] years; 52.0% women). Based on a 5% cutoff, the number of ADL and IADL limitations was higher in the groups of decreased (ADL: 0.56 [95% CI = 0.28, 0.85]; IADL: 0.66 [95% CI = 0.38, 0.94]) and increased BMI (ADL: 0.55 [95% CI = 0.28, 0.81]; IADL: 0.59 [95% CI = 0.33, 0.85]) compared to stable BMI, respectively. Similar findings were obtained for a 2% cutoff. These differences frequently persisted for 24 years for decreased BMI and 6–12 years for increased BMI.

**Conclusion:**

Early decreased BMI, and to a lesser extent increased BMI, following stroke could be a marker of long-term adverse trajectories of physical functioning, underlying the importance of nutritional and physical activity management after a stroke.

## 1 Introduction

Stroke is an acute event caused by vascular injury to the central nervous system, leading to a focal neurological deficit lasting more than 24 h or resulting in death within 24 h [1]. In 2019, there were approximately 101 million prevalent stroke cases worldwide [2]. Stroke is the second leading cause of death and the third leading cause of both death and disability. Stroke negatively impacts the quality of life of patients due to a range of factors, such as pain, depression, and inadequate social support [3]. In high-income countries, the mean annual cost per stroke survivor is 27,702 US dollars [4], with more than one-third of survivors having a post-stroke life expectancy of at least a decade [5]. The high economic burden related to stroke is explained by substantial direct and indirect costs [6]. A better understanding of factors associated with functional recovery after a stroke is critical to alleviating the adverse health and economic impact of stroke.

Weight loss is frequent following a stroke event. For example, in a study from Sweden, 26% of 305 patients with stroke had lost more than three kilograms at 12 months after stroke [7]. In recent years, there has been a growing interest in the effects of body mass index (BMI) on functional limitations and disability after stroke [[8], [9], [10], [11]]. Much of this research reports an association between BMI and functional outcomes (e.g., activities of daily living [ADLs] and instrumental activities of daily living [IADLs]) in adults with stroke, with, although it may seem counterintuitive, more favorable outcomes in those with overweight or obesity compared with normal weight individuals. A limitation in previous studies is a lack of data on the effects of BMI changes on functional outcome longitudinal trajectories after stroke. A single body of research has attempted to answer the question, showing that maintaining or gaining weight early after a stroke was associated with better functional outcomes than losing weight [12]. Nonetheless, this research should be viewed as preliminary, as the sample size was relatively small, as data were collected from only one institution, and as the study focused on hospitalized patients. In this context, given that stroke survivors are more vulnerable to accelerated rates of decline in functional limitations than the general population [13], investigating how BMI shapes these trajectories should be a public health priority.

Accordingly, using data from three cohorts conducted in Europe and the United States, this study examined whether changes in BMI post-stroke affect trajectories of functional limitations, measured here using ADLs and IADLs. The hypothesis was that decreased and increased BMI would be statistically associated with an increase in ADL and IADL difficulties, with the associations being stronger and more prolonged for decreased BMI.

## 2 Material and methods

### 2.1 Guidelines

This research followed the STROBE guidelines (cohort studies) (**Supplementary Table** 1).

### 2.2 Data

The present study was based on data from three cohort studies: the *Health and Retirement Study* (HRS) [14], the *Survey of Health, Ageing and Retirement in Europe* (SHARE) [15,16], and the *English Longitudinal Study of Ageing* (ELSA) [17]. HRS is a US nationally representative survey that was launched in 1992 and is conducted every 2 years. It includes adults living in the United States born in 1931–1941 at the 1992 inclusion and born in 1942–1947 for those included in 1998. SHARE is a survey conducted in 28 European countries and Israel, with a maximum of nine waves of data and with a wave launched approximately every 2 or 3 years. The first wave included adults born prior to 1955. Finally, ELSA, with a baseline in 2002, is a representative sample of adults born before 1953 and living in England. Participants were followed every 2 years for at least 11 waves. In all three cohort studies, the two major exclusion criteria were living in an institution and not being able to participate in the interviews or the assessments.

**Fig. 1:**
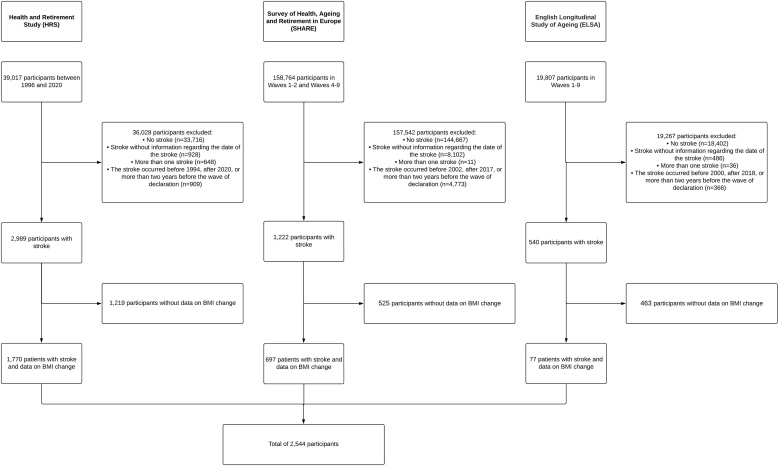
Flow chart of the study participants.

Participants included in the study were those with a first-ever stroke from 1994 to 2020 in HRS, 2002–2017 in SHARE, and 2000–2018 in ELSA. Participants with no data on the date of stroke, those with more than one stroke, those reporting a stroke that occurred outside the above years or more than two years before the wave of declaration, and those without data on BMI changes following stroke were excluded from the analyses. The study flow chart is shown in Fig. 1.

### 2.3 Stroke

Data on stroke were self-reported or reported by a proxy. In HRS, the question was, “Has a doctor ever told you that you had a stroke?”. In SHARE and ELSA, the respective question was, “Has a doctor ever told you that you had any of the conditions on this card [indicating a history of health condition]?”. Slight changes in the wording of the questions and the answers were possible.

### 2.4 Changes in BMI (independent variable)

BMI was defined as the ratio between weight (in kilograms) and height squared (in meters) [18]. BMI was self-reported every two years in HRS and SHARE (except for wave 9). BMI was objectively measured in ELSA by a nurse at waves 2 (2004–2005), 4 (2008–2009), and 6 (2012–2013) and self-reported at waves 8 (2016–2017) and 9 (2018–2019). When only weight was available for a given wave (i.e., 37.5% of the sample), height was imputed from the closest previous wave (i.e., within the last two years for 88.3% of cases).

Changes in BMI were defined as the percentage change in BMI between the wave at which the stroke was reported and the subsequent wave, which was two years later for 86.3% of the population. For those without data at year 2, changes in BMI were considered at year 1 (i.e., 0.5%), year 3 (i.e., 5.9%), and year 4 following stroke (7.4%). The data on changes in BMI were standardized for the number of years between the two BMI measures and categorized into three groups (decrease, stable, and increase in BMI) using 5% and then 2% change. The 5% cutoff has been used in the scientific literature [19], while the 2% cutoff represents a more stringent definition for BMI change.

### 2.5 Limitations in ADL and IADL (dependent variable)

Limitations in ADL [20] and IADL [21] were reported by the participant or their proxy over a median (interquartile range [IQR]) of 6.0 (7.0) and 6.0 (6.0) years after the wave they reported stroke. ADLs included bathing/showering, dressing, eating, getting in or out of bed, using the toilet, and walking across the room. IADLs included making a phone call, managing money, preparing a hot meal, shopping, taking medications, and using a map. For both scales, scores ranged from zero to six, with zero indicating autonomy and six total dependence.

### 2.6 Covariates

Covariates were drawn from the wave at which the stroke was reported and included age, sex, marital status, education level, alcohol consumption, current smoking, moderate-to-vigorous physical activity at least twice a week (only vigorous activity in HRS 1996–2002), number of chronic conditions (i.e., arthritis, asthma, cancer, cataracts, chronic lung disease, diabetes, heart condition, high blood pressure, high cholesterol, osteoporosis, and Parkinson’s disease), and BMI. In terms of education level, given that there was a substantial proportion of missing data, data were also retrieved from waves that preceded the wave of interest. The same analytical approach was applied to the number of comorbidities. For alcohol consumption, the time frame was lifetime for HRS, six months for SHARE wave 1, three months for SHARE waves 2, 4, and 5, seven days for SHARE waves 6, 7, 8, and 9, and 12 months for ELSA.

### 2.7 Statistical analysis

Data on covariates were missing on less than 5% of participants, except for current smoking, which had 13.8% missing data. Missing data was considered a separate category in the measure of current smoking. The main analyses were undertaken on complete cases.

Characteristics of the study population at inclusion were analyzed in the overall population and by BMI change categories based on the 5% and 2% thresholds. Categorical variables were described using N (%), numerical data with a normal distribution using the mean (standard deviation), and numerical data without a normal distribution using the median (IQR). P-values were obtained using Pearson’s chi-square, analysis of variance (ANOVA), and Kruskal-Wallis tests for categorical, numerical variables with a normal distribution, and numerical variables without a normal distribution, respectively.

The associations between changes in BMI and trajectories of ADLs and IADLs post-stroke were analyzed using segmented linear mixed-effects regression models. Given that BMI changes were mostly documented during the first two years of follow-up, the breakpoint between the two regression models was set at two years. This methodological approach allowed a distinction between the slope for short-term trajectories of ADL and IADL that are concurrent with BMI changes from long-term trajectories.

The regression models were adjusted for the BMI change categories, covariates (including baseline BMI), time, and the interactions of time with BMI changes and with covariates. Time was included as a linear term, as quadratic time was not statistically associated with the dependent variables. Age was included as a quadratic term; this term was always statistically significant. Besides the interaction between time and changes in BMI, other interactions with time were retained in the model only if they were statistically significant. The random-effects structure included a random intercept and a random slope for time. The assumptions for regression analysis (i.e., homoscedasticity, randomness of residuals, normality of residuals, and linearity of continuous predictors) were assessed graphically and found to hold.

The results (estimates and 95% confidence intervals [CI]) for the BMI change groups, extracted from the segmented linear mixed-effects regression, were differences in the number of ADL (and IADL, separate models) limitations at baseline (defined as the wave the participant reported stroke), the difference in changes in these limitations per year within the first two years of follow-up, and the difference in changes per year after two years of follow-up. In addition, the estimated marginal means of ADL/IADL limitations by BMI change categories were plotted over time. Differences in the number of ADL and IADL limitations every two years over the follow-up were also estimated.

Sensitivity analyses were conducted after imputing missing data using multiple imputation with chained equations. Missing data were imputed in each cohort study separately. Previous research has indicated that the number of datasets to impute should be equal to the proportion of participants with at least one missing data [22]. The highest proportion of individuals with at least one missing data was 50.4% across the three datasets. As preliminary analyses showed that a substantial proportion of models running on the imputed datasets did not converge, 100 datasets were imputed for each cohort study. In the final analyses, all models converged, and the 100 imputed datasets were used. Variables used to impute missing data were independent variables and data on ADL and IADL. In addition, as BMI and ADL/IADL limitations in the first two years of follow-up were measured concomitantly, we undertook additional analyses focusing on changes in the number of ADL and IADL limitations starting two years post-stroke. The number of ADL and IADL limitations at baseline was included as a covariate. These linear mixed-effects regression models did not include any breakpoints. As self-reported BMI may carry reporting biases [23], the concomitant use of self-reported and objectively measured BMI may affect results. To address this issue, we repeated the analyses excluding ELSA data that contained a mix of self-reported and measured BMI. The breakpoint set at two years may not reflect BMI changes that were measured at one, three, or four years. To address this issue, we repeated the analyses only on participants whose second BMI measure was exactly at two years. The analyses were repeated after including all interactions between time and the covariates to investigate their potential effects on the estimates.

Two-sided p-values <0.050 were considered statistically significant. All analyses were conducted using R version 4.4.2 [24].

## 3 Results

### 3.1 Baseline characteristics of the population

Overall, the study population included 2544 adults with stroke. The mean age (standard deviation) of participants was 70.0 (10.9) years at baseline, and 52.0% were women (Table 1). Compared to people with stable BMI, those with decreased or increased BMI were more likely to be women, not married or cohabiting, to have a primary education level, not to consume alcohol, to currently smoke, to be less physically active, and to have 2 or more comorbidities. Participants with decreased BMI were older and had higher BMI at stroke, and those with increased BMI were younger and had lower BMI at stroke compared with those with stable BMI. Participants were followed up for a maximum of 24 years.

**Table 1:** Baseline characteristics of the population in the overall sample and by BMI change.

Characteristics	Category	Overall (N = 2544)	BMI change, 5% cutoff				BMI change, 2% cutoff				Missing data
			Decreased (n = 230)	Stable (n = 2042)	Increased (n = 272)	P-value^a^	Decreased (n = 589)	Stable (n = 1269)	Increased (n = 686)	P-value^a^	
Age	Mean (SD)	70.0 (10.9)	71.1 (11.9)	70.0 (10.7)	69.3 (11.9)	<0.001	70.8 (11.3)	70.2 (10.5)	69.1 (11.2)	<0.001	1 (0.0)
Sex	Female	1324 (52.0)	151 (65.7)	1013 (49.6)	160 (58.8)	<0.001	348 (59.1)	590 (46.5)	386 (56.3)	<0.001	0 (0.0)
	Male	1220 (48.0)	79 (34.3)	1029 (50.4)	112 (41.2)		241 (40.9)	679 (53.5)	300 (43.7)		
Marital status	Single, separated, divorced, or widowed	1099 (43.3)	114 (49.6)	842 (41.4)	143 (52.6)	<0.001	272 (46.2)	522 (41.2)	305 (44.7)	<0.001	6 (0.2)
	Married or in a partnership	1439 (56.7)	116 (50.4)	1194 (58.6)	129 (47.4)		317 (53.8)	745 (58.8)	377 (55.3)		
Education level	Primary	757 (30.0)	73 (31.9)	584 (28.9)	100 (37.2)	<0.001	195 (33.3)	352 (28.0)	210 (30.8)	<0.001	23 (0.9)
	Secondary	949 (37.6)	76 (33.2)	781 (38.6)	92 (34.2)		207 (35.4)	475 (37.8)	267 (39.2)		
	Tertiary	815 (32.3)	80 (34.9)	658 (32.5)	77 (28.6)		183 (31.3)	428 (34.1)	204 (30.0)		
Alcohol consumption^b^	No	1439 (56.9)	155 (67.4)	1100 (54.3)	184 (67.9)	<0.001	363 (61.8)	649 (51.6)	427 (62.5)	<0.001	17 (0.7)
	Yes	1088 (43.1)	75 (32.6)	926 (45.7)	87 (32.1)		224 (38.2)	608 (48.4)	256 (37.5)		
Current smoking	No	1793 (81.7)	167 (79.9)	1440 (82.4)	186 (78.5)	<0.001	426 (81.5)	890 (82.9)	477 (79.9)	0.008	350 (13.8)
	Yes	401 (18.3)	42 (20.1)	308 (17.6)	51 (21.5)		97 (18.5)	184 (17.1)	120 (20.1)		
Moderate-to-vigorous physical activity^c^	No	1721 (67.8)	170 (73.9)	1341 (65.8)	210 (77.8)	<0.001	421 (71.6)	814 (64.3)	486 (71.2)	<0.001	7 (0.3)
	Yes	816 (32.2)	60 (26.1)	696 (34.2)	60 (22.2)		167 (28.4)	452 (35.7)	197 (28.8)		
Number of comorbidities^d^	0	801 (31.5)	43 (18.7)	686 (33.6)	72 (26.5)	<0.001	157 (26.7)	438 (34.5)	206 (30.0)	<0.001	0 (0.0)
	1	331 (13.0)	27 (11.7)	266 (13.0)	38 (14.0)		79 (13.4)	161 (12.7)	91 (13.3)		
	2 or more	1412 (55.5)	160 (69.6)	1090 (53.4)	162 (59.6)		353 (59.9)	670 (52.8)	389 (56.7)		
Body mass index	Median (IQR)	27.1 (6.4)	28.6 (7.8)	27.1 (6.1)	24.9 (6.8)	<0.001	28.5 (7.5)	27.1 (5.7)	25.8 (6.4)	<0.001	0 (0.0)
Continuous number of ADL limitations	Median (IQR)	0.0 (1.0)	1.0 (3.0)	0.0 (1.0)	1.0 (3.0)	<0.001	0.0 (2.0)	0.0 (1.0)	0.0 (2.0)	<0.001	0 (0.0)
Categorical number of ADL limitations	0	1578 (62.0)	107 (46.5)	1338 (65.5)	133 (48.9)	<0.001	327 (55.5)	838 (66.0)	413 (60.2)	<0.001	0 (0.0)
	1	341 (13.4)	37 (16.1)	264 (12.9)	40 (14.7)		88 (14.9)	171 (13.5)	82 (12.0)		
	2 or more	625 (24.6)	86 (37.4)	440 (21.5)	99 (36.4)		174 (29.5)	260 (20.5)	191 (27.8)		
Continuous number of IADL limitations	Median (IQR)	0.0 (2.0)	1.0 (4.0)	0.0 (2.0)	1.0 (4.0)	<0.001	1.0 (2.0)	0.0 (2.0)	1.0 (2.0)	<0.001	0 (0.0)
Categorical number of IADL limitations	0	1334 (52.4)	91 (39.6)	1142 (55.9)	101 (37.1)	<0.001	276 (46.9)	727 (57.3)	331 (48.3)	<0.001	0 (0.0)
	1	447 (17.6)	38 (16.5)	361 (17.7)	48 (17.6)		107 (18.2)	215 (16.9)	125 (18.2)		
	2 or more	763 (30.0)	101 (43.9)	539 (26.4)	123 (45.2)		206 (35.0)	327 (25.8)	230 (33.5)		

Abbreviations: ADL, activities of daily living; BMI, body mass index; IADL, instrumental activities of daily living; IQR, interquartile range; SD, standard deviation.

Data are N (%) unless otherwise stated.

a P-values were calculated using Pearson’s chi-square test for categorical variables, the analysis of variance test for age, and the Kruskal-Wallis test for BMI, ADL, and IADL at stroke.

b Alcohol consumption was assessed using different timeframes, which varied depending on the dataset and the wave.

c Moderate-to-vigorous physical activity was defined as doing moderate-to-vigorous physical activity at least twice a week.

d The comorbidities of interest were arthritis, asthma, cancer, cataracts, chronic lung disease, diabetes, heart condition, high blood pressure, high cholesterol, osteoporosis, and Parkinson’s disease.

### 3.2 Associations between changes in BMI and ADL trajectories after stroke

**Fig. 2:**
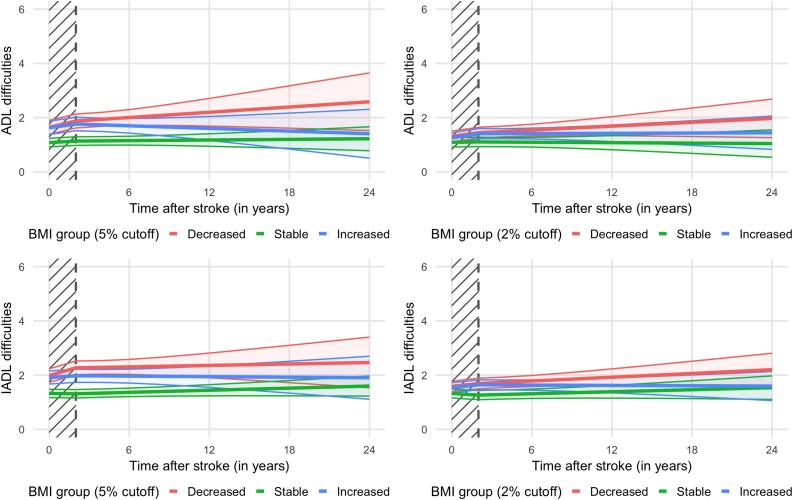
Trajectories of ADL and IADL limitations by BMI two-year change categories following stroke (upper panels: ADL limitations; lower panels: IADL limitations; left panels: 5% cutoff; right panels: 2% cutoff). Abbreviations: ADL, activities of daily living; BMI, body mass index; IADL, instrumental activities of daily living. The trajectories of ADL and IADL limitations by BMI changes after stroke were estimated using segmented linear mixed-effects regression models with the number of difficulties in ADLs or IADLs as the dependent variables, categories of BMI changes as the independent variable, with adjustment for age, sex, marital status, education level, alcohol consumption, current smoking, moderate-to-vigorous physical activity, the number of comorbidities, and body mass index (baseline covariates). The breakpoint was set at two years. ADLs and IADLs range from zero to six, with zero corresponding to full autonomy and six to full dependence. A hatch pattern background highlights the first two years of follow-up where BMI changes and ADL and IADL limitations were assessed concomitantly.

Using the 5% change cutoff, ADL limitations at baseline were higher in participants with decreased (estimate = 0.56 [95% CI = 0.28, 0.85]) and increased BMI (estimate = 0.55 [95% CI = 0.28, 0.81]) compared with the stable BMI group (Fig. 2 [upper panel] and Table 2). There was no significant difference in the increase in ADL limitations between the decreased or increased BMI group and the stable BMI group in the short-term follow-up (first two years) and the middle- and long-term follow-up (after two years post stroke). The results with the 2% cutoff were relatively similar, although the difference between the increased and the stable BMI group was no longer statistically significant at baseline (estimate = 0.16 [95% CI = −0.03, 0.35]). Regardless of the cutoff used (i.e., 5% or 2%), the differences between the changed BMI groups in the number of ADL limitations at baseline were maintained and remained statistically significant for 24 years for the decreased BMI group and 10 years for the increased BMI group (**Supplementary Table** 2). Results were relatively similar in the analyses based on the imputed data, although there were minor changes, with some differences in changes per year becoming statistically significant (**Supplementary Fig.** 1 [upper panels], **Supplementary Table** 3). In these analyses, the differences remained statistically significant for 24 years for decreased BMI compared with stable BMI, whereas the respective figures were eight (5% cutoff) and six years (2%) for increased BMI (**Supplementary Table** 4). The results of the sensitivity analyses, where changes in the number of ADL limitations were considered starting two years post-stroke, were similar to findings in the main analysis (**Supplementary Table** 5). The results were mostly unchanged by the exclusion of objectively measured BMI data (**Supplementary Table** 6) or changes in BMI that were not measured at exactly two years post-stroke (**Supplementary Table** 7). The inclusion of all interactions between time and covariates yielded similar estimates to those in the main analyses (**Supplementary Table** 8).

**Table 2:** Associations between change in BMI and trajectories of ADL limitations after stroke (segmented linear mixed-effects regression models).

Changes in BMI	Number of ADL limitations								
	Difference at baseline			Difference in changes per year during 0−2 years after stroke			Difference in changes per year after 2 years post-stroke		
	Estimate	95% CI	P-value	Estimate	95% CI	P-value	Estimate	95% CI	P-value
**5% cutoff**									
Decreased	0.56	(0.28, 0.85)	<0.001	0.09	(−0.03, 0.20)	0.209	0.03	(−0.03, 0.09)	0.504
Stable	Reference			Reference			Reference		
Increased	0.55	(0.28, 0.81)	<0.001	0.04	(−0.07, 0.15)	0.730	−0.02	(−0.07, 0.03)	0.504
**2% cutoff**									
Decreased	0.22	(0.02, 0.42)	0.029	0.06	(−0.03, 0.15)	0.186	0.03	(−0.02, 0.07)	0.379
Stable	Reference			Reference			Reference		
Increased	0.16	(−0.03, 0.35)	0.092	0.07	(−0.01, 0.15)	0.124	0.00	(−0.04, 0.04)	0.780

Abbreviations: ADL, activities of daily living; BMI, body mass index; CI, confidence interval.

Associations between change in BMI and trajectories of ADL limitations were estimated using segmented linear mixed-effects regression models adjusted for age, sex, marital status, education level, alcohol consumption, current smoking, moderate-to-vigorous physical activity, the number of comorbidities, and body mass index (baseline covariates).

The breakpoint was set at two years.

### 3.3 Associations between changes in BMI and IADL trajectories after stroke

Using the 5% cutoff, the decreased (estimate = 0.66 [95% CI = 0.38, 0.94]) and increased BMI groups (estimate = 0.59 [95% CI = 0.33, 0.85]) had more IADL limitations at baseline than the stable BMI group (Fig. 2 [lower panels] and Table 3). The decreased BMI group also had a faster increase in the number of IADL limitations over the short-term follow-up (first two years) compared with stable BMI (estimate = 0.15 [95% CI = 0.04, 0.26]). Results using the 2% cutoff to define BMI change were overall similar. The increase in the number of IADL limitations was also faster in the increased BMI than in the stable BMI group at the short-term follow-up (first two years) (estimate = 0.08 [95% CI = 0.00, 0.17]). Finally, the greater number of IADL limitations in the changed BMI groups compared with the stable BMI group observed at baseline remained statistically significant for 18 years for the decreased BMI group and 12 (5% cutoff) and eight years (2% cutoff) for the increased BMI group **(Supplementary Table** 9). The results of the analyses on the imputed data were similar (**Supplementary Fig.** 1 [lower panels], **Supplementary Table** 10, and **Supplementary Table** 11). The differences persisted for 24 years for both cutoffs for decreased BMI, while the respective figures were 10 (5%) and six years (2%) for increased BMI. The results were relatively similar for the models focusing on the number of IADL limitations starting two years after the wave at which stroke was declared (**Supplementary Table** 12). Neither the exclusion of objectively measured BMI data (**Supplementary Table** 13) nor the exclusion of BMI changes not measured at exactly two years post-stroke impacted the findings (**Supplementary Table** 14). The estimates were similar in the models including all interaction terms between time and the covariates (**Supplementary Table** 15).

**Table 3:** Associations between change in BMI and trajectories of IADL limitations after stroke (segmented linear mixed-effects regression models).

Changes in BMI	Number of IADL limitations								
	Difference at baseline			Difference in changes per year during 0−2 years after stroke			Difference in changes per year after 2 years post-stroke		
	Estimate	95% CI	P-value	Estimate	95% CI	P-value	Estimate	95% CI	P-value
**5% cutoff**									
Decreased	0.66	(0.38, 0.94)	<0.001	0.15	(0.04, 0.26)	0.005	−0.00	(−0.06, 0.05)	1.000
Stable	Reference			Reference			Reference		
Increased	0.59	(0.33, 0.85)	<0.001	0.04	(−0.07, 0.15)	0.404	−0.02	(−0.06, 0.03)	1.000
**2% cutoff**									
Decreased	0.26	(0.06, 0.47)	0.006	0.08	(−0.00, 0.17)	0.042	0.01	(−0.03, 0.05)	0.619
Stable	Reference			Reference			Reference		
Increased	0.21	(0.02, 0.40)	0.017	0.08	(0.00, 0.17)	0.040	−0.01	(−0.05, 0.02)	0.619

Abbreviations: BMI, body mass index; CI, confidence interval; IADL, instrumental activities of daily living.

Associations between change in BMI and trajectories of IADL limitations were estimated using segmented linear mixed-effects regression models adjusted for age, sex, marital status, education level, alcohol consumption, current smoking, moderate-to-vigorous physical activity, the number of comorbidities, and body mass index (baseline covariates).

The breakpoint was set at two years.

## 4 Discussion

### 4.1 Main findings

This study on 2544 participants from three cohort studies, followed for a maximum of 24 years after stroke, found 19.7% and 50.1% of participants reported BMI changes in the two years after stroke, using a 5% and 2% cutoff to define BMI changes, respectively. At baseline, participants with changes in BMI, particularly with a 5% change or more, had a greater number of ADL and IADL limitations. Although there were some differences in changes in the number of ADL and IADL limitations over the subsequent follow-up in the BMI change groups, there was no clear pattern in these changes. The differences observed at baseline between decreased and stable BMI were, most of the time, maintained for 24 years for both ADLs and IADLs, while the respective figure for increased BMI was 6–12 years.

### 4.2 Interpretation of the findings

The first study finding is that a substantial proportion of people had changes in BMI in the first two years following a stroke. This result is in line with previous studies that show decreases and increases in weight to be frequent in stroke survivors [7,25]. The decrease in weight can be explained by hemiplegia-related muscle mass loss [26], decreased physical activity [27], and increased sedentary behavior [28]. Although a decrease in muscle mass could partly explain weight loss, it is likely that weight loss following a stroke is a complex phenomenon that also involves other organs (e.g., the bone [29]). Post-stroke increase in weight could also be due to an increase in sedentary behavior and factors such as inadequate dietary intake [30] and the use of antidepressants [31].

The second key finding is that people with decreased or increased BMI had more limitations in ADLs and IADLs at baseline than their counterparts with stable BMI. Our focus was on change in BMI, measured as a continuous variable, rather than changes in obesity or overweight status that are based on categorization of BMI. There is some evidence to show that individuals with overweight and obesity have better functional outcomes after a stroke [[8], [9], [10], [11]]. Our study is not directly comparable, as our focus was on changes in BMI rather than overweight or obesity. The first mediating factor potentially involved in the associations observed in the study is sarcopenia, and stroke survivors with decreased BMI may be at higher risk of sarcopenia. A systematic review and meta-analysis of seven studies reported the prevalence of stroke-related sarcopenia to be 42%; the determinants of sarcopenia include older age, inadequate nutritional status, and bed rest [32]. More specifically, inadequate nutritional status can promote protein catabolism [33] and low-grade systemic inflammation [34] that could trigger sarcopenia. A longitudinal study on 6893 older adults followed over three years in China identified sarcopenia as a risk factor for ADL and IADL disability, the relationship potentially being mediated by reduced muscle strength and impaired physical functioning [35]. The association between decreased BMI and greater ADL and IADL limitations in stroke survivors could also involve myocardial infarction. Data obtained from more than 138,000 adults from the United Kingdom showed that those in the normal weight category who lost weight were at an increased risk for cardiovascular disease, including myocardial infarction [36]. Interestingly, hospitalization for myocardial infarction has also been identified as a risk factor for functional limitations in the following decade [37]. The effects of such a hospitalization on functional limitations could involve decreased cardiorespiratory fitness, impaired mobility, and reduced muscle strength. Similarly, cardiovascular diseases could also play a mediating role in the association between post-stroke increase in BMI and increase in the number of ADL and IADL limitations. Another potential mediator is back pain. Longitudinal analyses of ELSA data show a 5% increase in BMI to be associated with a 1.11-fold increase in the odds of back pain, and the effect size rose to 1.49 for a 25% increase in BMI [38]. As weight gain increases the load on the spine and its components (e.g., the disc), it could lead to its degeneration [39]. Another prospective cohort study of 754 older adults found an association between back pain and subsequent ADL and IADL disability [40]. Weight gain following stroke may also increase the risk of depression [41], which is itself known to increase the risk of functional disability [42].

A third major finding of the study is that, although BMI change was not clearly associated with changes in the number of ADL and IADL limitations during the follow-up, the baseline differences in the number of ADL and IADL difficulties between the BMI change groups persisted most of the time for 24 years for decreased BMI, and for 6–12 years for increased BMI. These findings should be interpreted with caution as BMI changes were only considered in the first two years after stroke. That being said, these prolonged and persistent associations call for an early management of weight after stroke. In addition, the more prolonged associations observed for decreased BMI compared with increased BMI likely highlight the fact that weight loss following a stroke may be the worst scenario from a functional perspective. Interestingly, similar conclusions have been obtained in a preliminary body of research that focused on the impact of one-month weight changes on function in 293 stroke survivors early after stroke [12].

### 4.3 Clinical implications and directions for future research

Our observational findings indicate that maintaining a stable BMI after stroke could be associated with lower odds of functional disability. These findings are in line with general guidelines on weight maintenance, particularly at older ages. Early identification of individuals with unstable weight at older ages may include assessing relevant biomarkers (e.g., albumin and prealbumin), monitoring appetite in patients, and evaluating mobility. Further research is needed to replicate our findings, particularly in settings where data on stroke subtype and severity are available to allow their role to be elucidated. Moreover, more research is required to identify physical activity tailored to the needs of this group of patients. Post-stroke depression is also frequent [43], and strategies to address depression are likely to involve a healthy diet and regular physical activity [44]. Further research is also needed on stroke survivors living in institutions, as they may be at higher risk for functional disability. Finally, studies based on objective measures of BMI and functional limitations would provide further insight into this association.

### 4.4 Strengths and limitations

The strengths of the study are the large sample size, data from several high-income countries, and the duration of follow-up. The study also has a few limitations. First, data were self-reported, which may have biased the findings. Although the specificity of self-reported measures of stroke is excellent (i.e., 96.0%–99.6%), the sensitivity ranges from 36.0% to 98.0% [45]. Self-reported BMI may carry some reporting bias [23], but it is likely that these biases are relatively constant in the same individuals over time and are unlikely to affect measures of change in BMI. Second, data on stroke subtype and stroke severity were not available, as is common in large longitudinal cohort studies. Both these measures are likely to be important, as the severity of stroke is known to be associated with both weight loss [7] and functional limitations [13]. Third, data were collected from the community, and the study findings cannot be generalized to stroke survivors living in institutions. A further limitation of our study is not being able to account for mortality in the analyses. Mortality rates may have been higher in BMI change groups and this is likely to affect estimates. Fourth, the 2% BMI change cutoff was arbitrarily chosen, and a different cutoff may have led to different results. Fifth, functional limitations were assessed based only on ADLs and IADLs, and the use of further functional outcomes may have allowed more detailed analyses.

### 4.5 Conclusions

This longitudinal study using data on 2544 older adults with stroke from Europe and the United States showed that BMI changes accompanying stroke were associated with a higher number of ADL and IADL limitations at baseline compared with stable BMI. This difference persisted for several years for both ADL and IADL limitations and was more pronounced for decreased BMI than for increased BMI.

## CRediT authorship contribution statement

Guillaume Chambinaud: conceptualization, formal analysis, funding acquisition, methodology, validation, visualization, writing – original draft, writing – review & editing. Aurore Fayosse: formal analysis, methodology, validation, visualization, writing – review & editing. Aline Dugravot: formal analysis, writing – review & editing. Benjamin Landré: writing – review & editing. Alexis Schnitzler: conceptualization, writing – review & editing. Archana Singh-Manoux: methodology, validation, visualization, writing – review & editing. Séverine Sabia: methodology, validation, visualization, writing – review & editing. Louis Jacob: conceptualization, formal analysis, funding acquisition, methodology, project administration, supervision, validation, visualization, writing – original draft, writing – review & editing.

## Ethics

The Institutional Review Board from the University of Michigan has provided ethical approval for the *Health and Retirement Study* (HRS). The Ethics Committee of the University of Mannheim has provided ethical approval for the *Survey of Health, Ageing and Retirement in Europe* (SHARE) study until 2018; the Ethics Council of the Max Planck Society provided ethical approval after 2018. Finally, the Research Ethics Committee from the National Health Service (NHS) provided ethical approval for the *English Longitudinal Study of Ageing* (ELSA).

## Presentation of the research

This research was presented as an abstract at a congress in France (“29èmes Journées de la Société Française Neuro-Vasculaire”).

## Declaration of Generative AI and AI-assisted technologies in the writing process

None.

## Funding sources

The HRS study is sponsored by the National Institute on Aging (grant number NIA U01AG009740) and is conducted by the University of Michigan. In addition, this paper uses data from SHARE Waves 1, 2, 4, 5, 6, 7, 8, 9 (DOIs: 10.6103/SHARE.w1.900, 10.6103/SHARE.w2.900, 10.6103/SHARE.w4.900, 10.6103/SHARE.w5.900, 10.6103/SHARE.w6.900, 10.6103/SHARE.w7.900, 10.6103/SHARE.w8.900, 10.6103/SHARE.w9.900). See Börsch-Supan et al. (2013) for methodological details. The SHARE data collection has been funded by the European Commission, DG RTD through <GN2>F</GN2>P5 (QLK6-CT-2001-00360), <GN2>F</GN2>P6 (SHARE-I3: RII-CT-2006-062193, <GN2>C</GN2>OMPARE: CIT5-CT-2005-028857, <GN2>S</GN2>HARELIFE: CIT4-CT-2006-028812), FP7 (SHARE-PREP: GA N°211909, <GN2>S</GN2>HARE-LEAP: GA N°227822, SHARE M4: GA N°261982,DASISH: GA N°283646) and Horizon 2020 (<GN2>S</GN2>HARE-DEV3: GA N°676536, <GN2>S</GN2>HARE-COHESION: GA N°870628, SERISS: GA N°654221,SSHOC: GA N°823782, <GN2>S</GN2>HARE-COVID19: GA N°101015924) and by DG Employment, Social Affairs & Inclusion through VS 2015/0195,VS 2016/0135,VS 2018/0285,VS 2019/0332,VS 2020/0313, <GN3>S</GN3>HARE-EUCOV: GA N°101052589 and EUCOVII: GA N°101102412. Additional funding from the German Federal Ministry of Education and Research (01UW1301,01UW1801,01UW2202), the Max Planck Society for the Advancement of Science, the U.S. National Institute on Aging (U01_AG09740-13S2,P01_AG005842,P01_AG08291,P30_AG12815,R21_AG025169,Y1-AG-4553-01,IAG_BSR06-11,OGHA_04-064,BSR12-04,R01_AG052527-02,R01_AG056329-02,R01_AG063944,HHSN271201300071C,RAG052527A) and from various national funding sources is gratefully acknowledged (see www.share-eric.eu). In addition, the study used data from ELSA, which is funded by the National Institute on Aging of the National Institutes of Health (award number: R01AG017644), and by UK Government Departments coordinated by the National Institute for Health and Care Research(NIHR; research program: 198_1074_03). The content is solely the responsibility of the authors and does not necessarily represent the official views of the funders of these three cohorts. Besides, we would like to thank the French Neurovascular Society (“Société Française de NeuroVasculaire”)for their financial support. Finally, this project is supported by a grant from “France 2030 ANR-23-PAVH-0006”.

## Data availability

The data can be accessed from the websites of the three respective studies after registration. The code is available from the corresponding author upon reasonable request.

## Declaration of competing interest

The authors have no conflict of interest to declare.

## Figures and Tables

**Figure:**
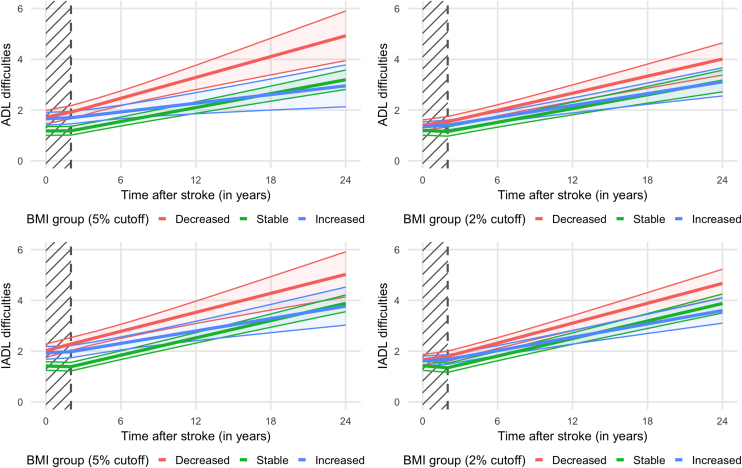


## Footnotes

Appendix A Supplementary material related to this article can be found, in the online version, at doi:https://doi.org/10.1016/j.jnha.2026.100772.
