# Supplementary material for: The association of post-stroke changes in body mass index with activity of daily living and instrumental activity of daily living trajectories: A multi-cohort analysis
Source: J Nutr Health Aging. 2026 Jan 12;30(2):100772. doi: 10.1016/j.jnha.2026.100772 (PMC12901523; doi:10.1016/j.jnha.2026.100772)
Supplement: Supplementary file 1 [file mmc1.docx]

**Supplementary Figure 1.** Trajectories of ADL and IADL limitations by BMI two-year change categories following stroke after imputing missing data using multiple imputation by chained equation (upper panels: ADL limitations; lower panels: IADL limitations; left panels: 5% cutoff; right panels: 2% cutoff)

Abbreviations: ADL, activities of daily living; BMI, body mass index; IADL instrumental activities of daily living.

The trajectories of ADL and IADL limitations by BMI changes after stroke were estimated using segmented linear mixed-effects regression models with the number of difficulties in ADLs or IADLs as the dependent variables, categories of BMI changes as the independent variable, with adjustment for age, sex, marital status, education level, alcohol consumption, current smoking, moderate-to-vigorous physical activity, the number of comorbidities, and body mass index (baseline covariates).

The breakpoint was set at two years.

ADLs and IADLs range from zero to six, with zero corresponding to full autonomy and six to full dependence.

A hatch pattern background highlights the first two years of follow-up where BMI changes and ADL and IADL limitations were assessed concomitantly.

**Supplementary Table 1.** STROBE Statement—Checklist of items that should be included in reports of cohort studies

|  | Item No | Recommendation | Page No |
| --- | --- | --- | --- |
| **Title and abstract** | 1 | (*a*) Indicate the study’s design with a commonly used term in the title or the abstract | Title and Abstract (Design) |
|  |  | (*b*) Provide in the abstract an informative and balanced summary of what was done and what was found | Abstract (Design, Setting, Participants, Measurements, and Results) |
| Introduction | | | |
| Background/rationale | 2 | Explain the scientific background and rationale for the investigation being reported | 1. Introduction (first and second paragraphs) |
| Objectives | 3 | State specific objectives, including any prespecified hypotheses | 1. Introduction (third paragraph) |
| Methods | | | |
| Study design | 4 | Present key elements of study design early in the paper | 2. Material and methods (2.2. Data) |
| Setting | 5 | Describe the setting, locations, and relevant dates, including periods of recruitment, exposure, follow-up, and data collection | 2. Material and methods (2.2. Data) |
| Participants | 6 | (*a*) Give the eligibility criteria, and the sources and methods of selection of participants. Describe methods of follow-up | 2. Material and methods (2.2. Data) |
|  |  | (*b*) For matched studies, give matching criteria and number of exposed and unexposed | Not applicable |
| Variables | 7 | Clearly define all outcomes, exposures, predictors, potential confounders, and effect modifiers. Give diagnostic criteria, if applicable | 2. Material and methods (2.3. Stroke, 2.4. Changes in BMI (independent variable), 2.5. Limitations in ADL and IADL (dependent variable), and 2.6. Covariates) |
| Data sources/ measurement | 8* | For each variable of interest, give sources of data and details of methods of assessment (measurement). Describe comparability of assessment methods if there is more than one group | 2. Material and methods (2.3. Stroke, 2.4. Changes in BMI (independent variable), 2.5. Limitations in ADL and IADL (dependent variable), and 2.6. Covariates) |
| Bias | 9 | Describe any efforts to address potential sources of bias | 2. Material and methods (2.7 Statistical analysis) |
| Study size | 10 | Explain how the study size was arrived at | 2. Material and methods (2.2. Data) and Figure 1 |
| Quantitative variables | 11 | Explain how quantitative variables were handled in the analyses. If applicable, describe which groupings were chosen and why | 2. Material and methods (2.7 Statistical analysis) |
| Statistical methods | 12 | (*a*) Describe all statistical methods, including those used to control for confounding | 2. Material and methods (2.7 Statistical analysis) |
|  |  | (*b*) Describe any methods used to examine subgroups and interactions | 2. Material and methods (2.7 Statistical analysis) |
|  |  | (*c*) Explain how missing data were addressed | 2. Material and methods (2.7 Statistical analysis) |
|  |  | (*d*) If applicable, explain how loss to follow-up was addressed | 2. Material and methods (2.7 Statistical analysis) |
|  |  | (*e*) Describe any sensitivity analyses | 2. Material and methods (2.7 Statistical analysis) |
| Results | | |  |
| Participants | 13* | (a) Report numbers of individuals at each stage of study—eg numbers potentially eligible, examined for eligibility, confirmed eligible, included in the study, completing follow-up, and analysed | Figure 1 |
|  |  | (b) Give reasons for non-participation at each stage | Figure 1 |
|  |  | (c) Consider use of a flow diagram | Figure 1 |
| Descriptive data | 14* | (a) Give characteristics of study participants (eg demographic, clinical, social) and information on exposures and potential confounders | 3. Results (3.1. Baseline characteristics of the population) and Table 1 |
|  |  | (b) Indicate number of participants with missing data for each variable of interest | Table 1 |
|  |  | (c) Summarise follow-up time (eg, average and total amount) | Figure 2 and Supplementary Figure 1 |
| Outcome data | 15* | Report numbers of outcome events or summary measures over time | 3. Results (3.2. Associations between changes in BMI and ADL trajectories after stroke and 3.3. Associations between changes in BMI and IADL trajectories after stroke), Figure 2, Supplementary Figure 1, Tables 2-3, and Supplementary Table 1-15 |

| Main results | 16 | (*a*) Give unadjusted estimates and, if applicable, confounder-adjusted estimates and their precision (eg, 95% confidence interval). Make clear which confounders were adjusted for and why they were included | Tables 2-3 and Supplementary Tables 1-15 |
| --- | --- | --- | --- |
|  |  | (*b*) Report category boundaries when continuous variables were categorized | Tables 2-3 and Supplementary Tables 1-15 |
|  |  | (*c*) If relevant, consider translating estimates of relative risk into absolute risk for a meaningful time period | Not applicable |
| Other analyses | 17 | Report other analyses done—eg analyses of subgroups and interactions, and sensitivity analyses | Supplementary Figure 1 and Supplementary Tables 1-15 |
| Discussion | | | |
| Key results | 18 | Summarise key results with reference to study objectives | 4. Discussion (4.1. Main findings) |
| Limitations | 19 | Discuss limitations of the study, taking into account sources of potential bias or imprecision. Discuss both direction and magnitude of any potential bias | 4. Discussion (4.4. Strengths and limitations) |
| Interpretation | 20 | Give a cautious overall interpretation of results considering objectives, limitations, multiplicity of analyses, results from similar studies, and other relevant evidence | 4. Discussion (4.2. Interpretation of the findings) |
| Generalisability | 21 | Discuss the generalisability (external validity) of the study results | 4. Discussion (4.2. Interpretation of the findings) and 4. Discussion (4.3. Clinical implications and directions for future research) |
| Other information | | | |
| Funding | 22 | Give the source of funding and the role of the funders for the present study and, if applicable, for the original study on which the present article is based | Declarations (Funding sources) |

*Give information separately for exposed and unexposed groups.

**Note:** An Explanation and Elaboration article discusses each checklist item and gives methodological background and published examples of transparent reporting. The STROBE checklist is best used in conjunction with this article (freely available on the Web sites of PLoS Medicine at http://www.plosmedicine.org/, Annals of Internal Medicine at http://www.annals.org/, and Epidemiology at http://www.epidem.com/). Information on the STROBE Initiative is available at http://www.strobe-statement.org.

**Supplementary Table 2.** Differences in the number of ADL limitations by two-year BMI change in the years following stroke

| **Time (in years)** | **Difference in the number of ADL limitations when comparing groups of:** | | | | | |
| --- | --- | --- | --- | --- | --- | --- |
|  | **Decreased versus stable BMI** | | | **Increased versus stable BMI** | | |
|  | **Estimate** | **95% CI** | **P-value** | **Estimate** | **95% CI** | **P-value** |
| **5% cutoff** | | | | | | |
| 0 | 0.56 | (0.28, 0.85) | <0.001 | 0.55 | (0.28, 0.81) | <0.001 |
| 2 | 0.74 | (0.46, 1.02) | <0.001 | 0.63 | (0.36, 0.90) | <0.001 |
| 4 | 0.80 | (0.51, 1.08) | <0.001 | 0.59 | (0.32, 0.86) | <0.001 |
| 6 | 0.85 | (0.52, 1.19) | <0.001 | 0.55 | (0.24, 0.86) | <0.001 |
| 8 | 0.91 | (0.50, 1.32) | <0.001 | 0.51 | (0.14, 0.88) | 0.002 |
| 10 | 0.97 | (0.46, 1.47) | <0.001 | 0.47 | (0.02, 0.92) | 0.026 |
| 12 | 1.02 | (0.42, 1.63) | <0.001 | 0.43 | (-0.11, 0.96) | 0.113 |
| 14 | 1.08 | (0.37, 1.80) | <0.001 | 0.39 | (-0.24, 1.02) | 0.140 |
| 16 | 1.14 | (0.31, 1.96) | 0.003 | 0.35 | (-0.38, 1.07) | 0.251 |
| 18 | 1.19 | (0.26, 2.13) | 0.007 | 0.31 | (-0.51, 1.13) | 0.371 |
| 20 | 1.25 | (0.20, 2.31) | 0.014 | 0.27 | (-0.65, 1.18) | 0.488 |
| 22 | 1.31 | (0.14, 2.48) | 0.022 | 0.23 | (-0.79, 1.24) | 0.595 |
| 24 | 1.37 | (0.08, 2.65) | 0.033 | 0.19 | (-0.93, 1.30) | 0.692 |
| **2% cutoff** | | | | | | |
| 0 | 0.22 | (0.02, 0.42) | 0.029 | 0.16 | (-0.03, 0.35) | 0.092 |
| 2 | 0.34 | (0.13, 0.55) | <0.001 | 0.30 | (0.10, 0.50) | <0.001 |
| 4 | 0.39 | (0.18, 0.61) | <0.001 | 0.31 | (0.11, 0.51) | <0.001 |
| 6 | 0.45 | (0.21, 0.69) | <0.001 | 0.32 | (0.09, 0.55) | 0.002 |
| 8 | 0.50 | (0.21, 0.80) | <0.001 | 0.33 | (0.05, 0.60) | 0.008 |
| 10 | 0.55 | (0.19, 0.91) | <0.001 | 0.34 | (0.01, 0.67) | 0.030 |
| 12 | 0.61 | (0.18, 1.04) | 0.002 | 0.35 | (-0.05, 0.74) | 0.072 |
| 14 | 0.66 | (0.15, 1.17) | 0.005 | 0.35 | (-0.11, 0.82) | 0.133 |
| 16 | 0.72 | (0.24, 1.19) | 0.010 | 0.36 | (-0.07, 0.80) | 0.204 |
| 18 | 0.77 | (0.11, 1.43) | 0.017 | 0.37 | (-0.23, 0.98) | 0.279 |
| 20 | 0.82 | (0.08, 1.57) | 0.025 | 0.38 | (-0.29, 1.06) | 0.353 |
| 22 | 0.88 | (0.05, 1.70) | 0.033 | 0.39 | (-0.36, 1.14) | 0.422 |
| 24 | 0.93 | (0.02, 1.83) | 0.042 | 0.40 | (-0.42, 1.22) | 0.427 |

Abbreviations: ADL, activities of daily living; BMI, body mass index; CI, confidence interval.

Differences in the number of ADL limitations by BMI changes were estimated using segmented linear mixed-effects regression models adjusted for age, sex, marital status, education level, alcohol consumption, current smoking, moderate-to-vigorous physical activity, the number of comorbidities, and body mass index (baseline covariates).

**Supplementary Table 3.** Associations between change in BMI and trajectories of ADL limitations after stroke after imputing missing data using multiple imputation by chained equation (segmented linear mixed-effects regression models)

|  | **Number of ADL limitations** | | | | | | | | |
| --- | --- | --- | --- | --- | --- | --- | --- | --- | --- |
| **Changes in BMI** | **Difference at baseline** | | | **Difference in changes per year during 0-2 years after stroke** | | | **Difference in changes per year after 2 years post-stroke** | | |
|  | **Estimate** | **95% CI** | **P-value** | **Estimate** | **95% CI** | **P-value** | **Estimate** | **95% CI** | **P-value** |
| **5% cutoff** | | | | | | | | | |
| Decreased | 0.55 | (0.32, 0.77) | <0.001 | 0.09 | (0.01, 0.18) | 0.034 | 0.05 | (0.00, 0.09) | 0.042 |
| Stable | Reference | | | Reference | | | Reference | | |
| Increased | 0.48 | (0.27, 0.69) | <0.001 | 0.02 | (-0.06, 0.10) | 0.693 | -0.03 | (-0.07, 0.00) | 0.067 |
| **2% cutoff** | | | | | | | | | |
| Decreased | 0.21 | (0.04, 0.37) | 0.015 | 0.09 | (0.03, 0.15) | 0.005 | 0.02 | (-0.05, 0.01) | 0.148 |
| Stable | Reference | | | Reference | | | Reference | | |
| Increased | 0.14 | (-0.01, 0.30) | 0.073 | 0.05 | (0.00, 0.11) | 0.068 | -0.01 | (-0.04, 0.01) | 0.331 |

Abbreviations: ADL, activities of daily living; BMI, body mass index; CI, confidence interval; SHARE, *Survey of Health, Ageing and Retirement in Europe*.

Associations between change in BMI and trajectories of ADL limitations were estimated using segmented linear mixed-effects regression models adjusted for age, sex, marital status, education level, alcohol consumption, current smoking, moderate-to-vigorous physical activity, the number of comorbidities, and body mass index (baseline covariates).

The breakpoint was set at two years.

Missing data were imputed using multiple imputation by chained equation, and 100 datasets were imputed. The number of imputed datasets corresponded to approximately twice the highest proportion of participants with at least one missing data, which was found in the SHARE dataset (i.e., 50.4%).

**Supplementary Table 4.** Differences in the number of ADL limitations by two-year BMI change in the years following stroke after imputing missing data using multiple imputation by chained equation (segmented linear mixed-effects regression models)

| **Time (in years)** | **Difference in the number of ADL limitations when comparing groups of:** | | | | | |
| --- | --- | --- | --- | --- | --- | --- |
|  | **Decreased versus stable BMI** | | | **Increased versus stable BMI** | | |
|  | **Estimate** | **95% CI** | **P-value** | **Estimate** | **95% CI** | **P-value** |
| **5% cutoff** | | | | | | |
| 0 | 0.55 | (0.32, 0.77) | <0.001 | 0.48 | (0.27, 0.69) | <0.001 |
| 2 | 0.73 | (0.51, 0.95) | <0.001 | 0.51 | (0.31, 0.72) | <0.001 |
| 4 | 0.82 | (0.60, 1.05) | <0.001 | 0.45 | (0.24, 0.65) | <0.001 |
| 6 | 0.91 | (0.66, 1.17) | <0.001 | 0.38 | (0.15, 0.61) | 0.001 |
| 8 | 1.01 | (0.70, 1.32) | <0.001 | 0.31 | (0.03, 0.58) | 0.027 |
| 10 | 1.10 | (0.72, 1.47) | <0.001 | 0.24 | (-0.09, 0.57) | 0.151 |
| 12 | 1.19 | (0.74, 1.64) | <0.001 | 0.17 | (-0.22, 0.56) | 0.387 |
| 14 | 1.28 | (0.75, 1.81) | <0.001 | 0.10 | (-0.35, 0.55) | 0.657 |
| 16 | 1.37 | (0.76, 1.98) | <0.001 | 0.03 | (-0.48, 0.55) | 0.898 |
| 18 | 1.46 | (0.77, 2.15) | <0.001 | -0.03 | (-0.62, 0.55) | 0.907 |
| 20 | 1.55 | (0.77, 2.33) | <0.001 | -0.10 | (-0.76, 0.55) | 0.757 |
| 22 | 1.64 | (0.78, 2.50) | <0.001 | -0.17 | (-0.90, 0.55) | 0.643 |
| 24 | 1.73 | (0.79, 2.68) | <0.001 | -0.24 | (-1.04, 0.56) | 0.554 |
| **2% cutoff** | | | | | | |
| 0 | 0.21 | (0.04, 0.37) | 0.015 | 0.14 | (-0.01, 0.30) | 0.073 |
| 2 | 0.39 | (0.23, 0.55) | <0.001 | 0.25 | (0.10, 0.40) | <0.001 |
| 4 | 0.43 | (0.27, 0.59) | <0.001 | 0.23 | (0.08, 0.38) | 0.003 |
| 6 | 0.47 | (0.29, 0.65) | <0.001 | 0.20 | (0.03, 0.37) | 0.019 |
| 8 | 0.52 | (0.30, 0.73) | <0.001 | 0.17 | (-0.02, 0.37) | 0.083 |
| 10 | 0.56 | (0.30, 0.82) | <0.001 | 0.15 | (-0.09, 0.38) | 0.217 |
| 12 | 0.60 | (0.30, 0.91) | <0.001 | 0.12 | (-0.16, 0.40) | 0.389 |
| 14 | 0.65 | (0.29, 1.01) | <0.001 | 0.10 | (-0.23, 0.42) | 0.561 |
| 16 | 0.69 | (0.28, 1.11) | 0.001 | 0.07 | (-0.30, 0.44) | 0.711 |
| 18 | 0.73 | (0.27, 1.20) | 0.002 | 0.04 | (-0.38, 0.47) | 0.837 |
| 20 | 0.78 | (0.25, 1.30) | 0.004 | 0.02 | (-0.45, 0.49) | 0.940 |
| 22 | 0.82 | (0.24, 1.40) | 0.006 | -0.01 | (-0.53, 0.51) | 0.976 |
| 24 | 0.87 | (0.23, 1.51) | 0.008 | -0.03 | (-0.61, 0.54) | 0.907 |

Abbreviations: ADL, activities of daily living; BMI, body mass index; CI, confidence interval; SHARE, *Survey of Health, Ageing and Retirement in Europe*.

Differences in the number of ADL limitations by BMI changes were estimated using segmented linear mixed-effects regression models adjusted for age, sex, marital status, education level, alcohol consumption, current smoking, moderate-to-vigorous physical activity, the number of comorbidities, and body mass index (baseline covariates).

Missing data were imputed using multiple imputation by chained equation, and 100 datasets were imputed. The number of imputed datasets corresponded to approximately twice the highest proportion of participants with at least one missing data, which was found in the SHARE dataset (i.e., 50.4%).

**Supplementary Table 5.** Associations between change in BMI and trajectories of ADL limitations starting two years after stroke (linear mixed-effects regression models)

| **Number of ADL limitations** | | | | | | |
| --- | --- | --- | --- | --- | --- | --- |
| **Changes in BMI** | **Difference at two years** | | | **Difference in changes per year after 2 years post-stroke** | | |
|  | **Estimate** | **95% CI** | **P-value** | **Estimate** | **95% CI** | **P-value** |
| **5% cutoff** | | | | | | |
| Decreased | 0.32 | (0.10, 0.53) | 0.002 | 0.03 | (-0.03, 0.09) | 0.374 |
| Stable | Reference | | | Reference | | |
| Increased | 0.20 | (-0.01, 0.41) | 0.046 | -0.02 | (-0.07, 0.03) | 0.374 |
| **2% cutoff** | | | | | | |
| Decreased | 0.34 | (0.10, 0.57) | 0.002 | 0.02 | (-0.02, 0.06) | 0.575 |
| Stable | Reference | | | Reference | | |
| Increased | 0.24 | (0.03, 0.47) | 0.018 | -0.00 | (-0.04, 0.04) | 0.935 |

Abbreviations: ADL, activities of daily living; BMI, body mass index; CI, confidence interval.

Associations between change in BMI and trajectories of ADL limitations were estimated using segmented linear mixed-effects regression models adjusted for age, sex, marital status, education level, alcohol consumption, current smoking, moderate-to-vigorous physical activity, the number of comorbidities, body mass index, and the number of ADL limitations (baseline covariates).

The follow-up started two years after the wave at which stroke was declared. No breakpoint was used for this model.

**Supplementary Table 6.** Associations between change in BMI and trajectories of ADL limitations after stroke (segmented linear mixed-effects regression models based on self-reported measures of BMI only)

|  | **Number of ADL limitations** | | | | | | | | |
| --- | --- | --- | --- | --- | --- | --- | --- | --- | --- |
| **Changes in BMI** | **Difference at baseline** | | | **Difference in changes per year during 0-2 years after stroke** | | | **Difference in changes per year after 2 years post-stroke** | | |
|  | **Estimate** | **95% CI** | **P-value** | **Estimate** | **95% CI** | **P-value** | **Estimate** | **95% CI** | **P-value** |
| **5% cutoff** | | | | | | | | | |
| Decreased | 0.56 | (0.28, 0.84) | <0.001 | 0.09 | (-0.03, 0.20) | 0.225 | 0.02 | (-0.04, 0.08) | 0.518 |
| Stable | Reference | | | Reference | | | Reference | | |
| Increased | 0.54 | (0.28, 0.81) | <0.001 | 0.04 | (-0.07, 0.16) | 0.730 | -0.02 | (-0.08, 0.03) | 0.518 |
| **2% cutoff** | | | | | | | | | |
| Decreased | 0.22 | (0.02, 0.43) | 0.030 | 0.06 | (-0.03, 0.15) | 0.214 | 0.02 | (-0.02, 0.07) | 0.587 |
| Stable | Reference | | | Reference | | | Reference | | |
| Increased | 0.17 | (-0.03, 0.37) | 0.081 | 0.07 | (-0.02, 0.15) | 0.167 | 0.00 | (-0.04, 0.04) | 0.981 |

Abbreviations: ADL, activities of daily living; BMI, body mass index; CI, confidence interval; HRS, *Health and Retirement Study*; SHARE, *Survey of Health, Ageing and Retirement in Europe*.

Associations between change in BMI and trajectories of ADL limitations were estimated using segmented linear mixed-effects regression models adjusted for age, sex, marital status, education level, alcohol consumption, current smoking, moderate-to-vigorous physical activity, the number of comorbidities, and body mass index (baseline covariates).

The breakpoint was set at two years.

This sensitivity analysis was based on HRS and SHARE data that corresponded to self-reported measures of BMI.

**Supplementary Table 7.** Associations between change in BMI measured exactly at two years and trajectories of ADL limitations after stroke (segmented linear mixed-effects regression models)

|  | **Number of ADL limitations** | | | | | | | | |
| --- | --- | --- | --- | --- | --- | --- | --- | --- | --- |
| **Changes in BMI** | **Difference at baseline** | | | **Difference in changes per year during 0-2 years after stroke** | | | **Difference in changes per year after 2 years post-stroke** | | |
|  | **Estimate** | **95% CI** | **P-value** | **Estimate** | **95% CI** | **P-value** | **Estimate** | **95% CI** | **P-value** |
| **5% cutoff** | | | | | | | | | |
| Decreased | 0.57 | (0.28, 0.86) | <0.001 | 0.08 | (-0.03, 0.20) | 0.251 | 0.02 | (-0.04, 0.08) | 0.699 |
| Stable | Reference | | | Reference | | | Reference | | |
| Increased | 0.52 | (0.25, 0.80) | <0.001 | 0.04 | (-0.07, 0.15) | 0.776 | -0.02 | (-0.08, 0.03) | 0.699 |
| **2% cutoff** | | | | | | | | | |
| Decreased | 0.20 | (-0.02, 0.42) | 0.081 | 0.06 | (-0.03, 0.15) | 0.196 | 0.03 | (-0.02, 0.07) | 0.471 |
| Stable | Reference | | | Reference | | | Reference | | |
| Increased | 0.12 | (-0.09, 0.33) | 0.336 | 0.07 | (-0.01, 0.16) | 0.108 | 0.00 | (-0.04, 0.04) | 0.887 |

Abbreviations: ADL, activities of daily living; BMI, body mass index; CI, confidence interval.

Associations between change in BMI and trajectories of ADL limitations were estimated using segmented linear mixed-effects regression models adjusted for age, sex, marital status, education level, alcohol consumption, current smoking, moderate-to-vigorous physical activity, the number of comorbidities, and body mass index (baseline covariates).

The breakpoint was set at two years.

In these analyses, BMI change was measured at two years exactly, and measures obtained at one, three, and four years were excluded.

**Supplementary Table 8.** Associations between change in BMI and trajectories of ADL limitations after stroke (segmented linear mixed-effects regression models including all interactions between time and the covariates)

|  | **Number of ADL limitations** | | | | | | | | |
| --- | --- | --- | --- | --- | --- | --- | --- | --- | --- |
| **Changes in BMI** | **Difference at baseline** | | | **Difference in changes per year during 0-2 years after stroke** | | | **Difference in changes per year after 2 years post-stroke** | | |
|  | **Estimate** | **95% CI** | **P-value** | **Estimate** | **95% CI** | **P-value** | **Estimate** | **95% CI** | **P-value** |
| **5% cutoff** | | | | | | | | | |
| Decreased | 0.56 | (0.28, 0.85) | <0.001 | 0.09 | (-0.03, 0.20) | 0.233 | 0.03 | (-0.03, 0.09) | 0.499 |
| Stable | Reference | | | Reference | | | Reference | | |
| Increased | 0.55 | (0.29, 0.81) | <0.001 | 0.04 | (-0.07, 0.15) | 0.793 | -0.02 | (-0.07, 0.03) | 0.499 |
| **2% cutoff** | | | | | | | | | |
| Decreased | 0.22 | (0.02, 0.42) | 0.030 | 0.06 | (-0.03, 0.15) | 0.199 | 0.03 | (-0.02, 0.07) | 0.414 |
| Stable | Reference | | | Reference | | | Reference | | |
| Increased | 0.16 | (-0.03, 0.36) | 0.087 | 0.07 | (-0.01, 0.15) | 0.134 | 0.00 | (-0.04, 0.04) | 0.833 |

Abbreviations: ADL, activities of daily living; BMI, body mass index; CI, confidence interval.

Associations between change in BMI and trajectories of ADL limitations were estimated using segmented linear mixed-effects regression models adjusted for age, sex, marital status, education level, alcohol consumption, current smoking, moderate-to-vigorous physical activity, the number of comorbidities, and body mass index (baseline covariates).

The breakpoint was set at two years.

These analyses included all interactions between time, which was linear, and the covariates.

**Supplementary Table 9.** Differences in the number of IADL limitations by two-year BMI change in the years following stroke

| **Time (in years)** | **Difference in the number of IADL limitations when comparing groups of:** | | | | | |
| --- | --- | --- | --- | --- | --- | --- |
|  | **Decreased versus stable BMI** | | | **Increased versus stable BMI** | | |
|  | **Estimate** | **95% CI** | **P-value** | **Estimate** | **95% CI** | **P-value** |
| **5% cutoff** | | | | | | |
| 0 | 0.66 | (0.38, 0.94) | <0.001 | 0.59 | (0.33, 0.85) | <0.001 |
| 2 | 0.96 | (0.68, 1.24) | <0.001 | 0.66 | (0.40, 0.93) | <0.001 |
| 4 | 0.95 | (0.67, 1.22) | <0.001 | 0.63 | (0.37, 0.89) | <0.001 |
| 6 | 0.94 | (0.63, 1.25) | <0.001 | 0.60 | (0.31, 0.89) | <0.001 |
| 8 | 0.93 | (0.55, 1.31) | <0.001 | 0.57 | (0.22, 0.91) | <0.001 |
| 10 | 0.92 | (0.46, 1.38) | <0.001 | 0.53 | (0.13, 0.94) | 0.004 |
| 12 | 0.92 | (0.37, 1.47) | <0.001 | 0.50 | (0.02, 0.99) | 0.026 |
| 14 | 0.91 | (0.26, 1.55) | 0.002 | 0.47 | (-0.10, 1.03) | 0.093 |
| 16 | 0.90 | (0.15, 1.65) | 0.012 | 0.44 | (-0.21, 1.09) | 0.214 |
| 18 | 0.89 | (0.04, 1.74) | 0.035 | 0.40 | (-0.33, 1.14) | 0.375 |
| 20 | 0.88 | (-0.07, 1.83) | 0.078 | 0.37 | (-0.45, 1.19) | 0.557 |
| 22 | 0.88 | (-0.18, 1.93) | 0.140 | 0.34 | (-0.57, 1.25) | 0.658 |
| 24 | 0.87 | (-0.29, 2.03) | 0.219 | 0.31 | (-0.69, 1.31) | 0.706 |
| **2% cutoff** | | | | | | |
| 0 | 0.26 | (0.06, 0.47) | 0.006 | 0.21 | (0.02, 0.40) | 0.017 |
| 2 | 0.43 | (0.22, 0.63) | <0.001 | 0.38 | (0.18, 0.58) | <0.001 |
| 4 | 0.45 | (0.24, 0.65) | <0.001 | 0.35 | (0.16, 0.54) | <0.001 |
| 6 | 0.47 | (0.24, 0.70) | <0.001 | 0.32 | (0.11, 0.54) | <0.001 |
| 8 | 0.49 | (0.22, 0.76) | <0.001 | 0.29 | (0.04, 0.55) | 0.012 |
| 10 | 0.51 | (0.19, 0.84) | <0.001 | 0.26 | (-0.04, 0.57) | 0.075 |
| 12 | 0.54 | (0.15, 0.92) | 0.003 | 0.23 | (-0.12, 0.59) | 0.198 |
| 14 | 0.56 | (0.10, 1.01) | 0.010 | 0.20 | (-0.21, 0.62) | 0.241 |
| 16 | 0.58 | (0.05, 1.10) | 0.025 | 0.18 | (-0.30, 0.65) | 0.382 |
| 18 | 0.60 | (0.01, 1.19) | 0.047 | 0.15 | (-0.40, 0.69) | 0.520 |
| 20 | 0.62 | (-0.04, 1.29) | 0.077 | 0.12 | (-0.49, 0.72) | 0.645 |
| 22 | 0.64 | (-0.10, 1.38) | 0.111 | 0.09 | (-0.59, 0.76) | 0.755 |
| 24 | 0.66 | (-0.15, 1.47) | 0.150 | 0.06 | (-0.68, 0.80) | 0.850 |

Abbreviations: BMI, body mass index; CI, confidence interval; IADL, activities of daily living.

Differences in the number of IADL limitations by BMI changes were estimated using segmented linear mixed-effects regression models adjusted for age, sex, marital status, education level, alcohol consumption, current smoking, moderate-to-vigorous physical activity, the number of comorbidities, and body mass index (baseline covariates).

**Supplementary Table 10.** Associations between change in BMI and trajectories of IADL limitations after stroke after imputing missing data using multiple imputation by chained equation (segmented linear mixed-effects regression models)

|  | **Number of IADL limitations** | | | | | | | | |
| --- | --- | --- | --- | --- | --- | --- | --- | --- | --- |
| **Changes in BMI** | **Difference at baseline** | | | **Difference in changes per year during 0-2 years after stroke** | | | **Difference in changes per year after 2 years post-stroke** | | |
|  | **Estimate** | **95% CI** | **P-value** | **Estimate** | **95% CI** | **P-value** | **Estimate** | **95% CI** | **P-value** |
| **5% cutoff** | | | | | | | | | |
| Decreased | 0.60 | (0.38, 0.83) | <0.001 | 0.15 | (0.06, 0.23) | 0.001 | 0.01 | (-0.05, 0.03) | 0.591 |
| Stable | Reference | | | Reference | | | Reference | | |
| Increased | 0.52 | (0.31, 0.73) | <0.001 | 0.04 | (-0.04, 0.12) | 0.374 | -0.03 | (-0.07, 0.00) | 0.069 |
| **2% cutoff** | | | | | | | | | |
| Decreased | 0.23 | (0.07, 0.40) | 0.006 | 0.11 | (0.05, 0.17) | <0.001 | 0.02 | (-0.01, 0.04) | 0.268 |
| Stable | Reference | | | Reference | | | Reference | | |
| Increased | 0.19 | (0.03, 0.35) | 0.018 | 0.07 | (0.01, 0.13) | 0.023 | -0.03 | (-0.05, -0.00) | 0.029 |

Abbreviations: BMI, body mass index; CI, confidence interval; IADL, instrumental activities of daily living; SHARE, *Survey of Health, Ageing and Retirement in Europe*.

Associations between change in BMI and trajectories of IADL limitations were estimated using segmented linear mixed-effects regression models adjusted for age, sex, marital status, education level, alcohol consumption, current smoking, moderate-to-vigorous physical activity, the number of comorbidities, and body mass index (baseline covariates).

The breakpoint was set at two years.

Missing data were imputed using multiple imputation by chained equation, and 100 datasets were imputed. The number of imputed datasets corresponded to approximately twice the highest proportion of participants with at least one missing data, which was found in the SHARE dataset (i.e., 50.4%).

**Supplementary Table 11.** Differences in the number of IADL limitations by two-year BMI change in the years following stroke after imputing missing data using multiple imputation by chained equation (segmented linear mixed-effects regression models)

| **Time (in years)** | **Difference in the number of IADL limitations when comparing groups of:** | | | | | |
| --- | --- | --- | --- | --- | --- | --- |
|  | **Decreased versus stable BMI** | | | **Increased versus stable BMI** | | |
|  | **Estimate** | **95% CI** | **P-value** | **Estimate** | **95% CI** | **P-value** |
| **5% cutoff** | | | | | | |
| 0 | 0.60 | (0.38, 0.83) | <0.001 | 0.52 | (0.31, 0.73) | <0.001 |
| 2 | 0.89 | (0.68, 1.11) | <0.001 | 0.59 | (0.39, 0.79) | <0.001 |
| 4 | 0.92 | (0.70, 1.13) | <0.001 | 0.53 | (0.33, 0.73) | <0.001 |
| 6 | 0.94 | (0.70, 1.18) | <0.001 | 0.46 | (0.24, 0.68) | <0.001 |
| 8 | 0.96 | (0.67, 1.25) | <0.001 | 0.40 | (0.14, 0.66) | 0.002 |
| 10 | 0.98 | (0.63, 1.34) | <0.001 | 0.34 | (0.03, 0.64) | 0.032 |
| 12 | 1.01 | (0.58, 1.43) | <0.001 | 0.27 | (-0.09, 0.63) | 0.140 |
| 14 | 1.03 | (0.53, 1.52) | <0.001 | 0.21 | (-0.21, 0.63) | 0.330 |
| 16 | 1.05 | (0.48, 1.62) | <0.001 | 0.14 | (-0.34, 0.63) | 0.555 |
| 18 | 1.07 | (0.43, 1.72) | 0.001 | 0.08 | (-0.46, 0.63) | 0.769 |
| 20 | 1.10 | (0.37, 1.82) | 0.002 | 0.02 | (-0.59, 0.63) | 0.954 |
| 22 | 1.12 | (0.32, 1.92) | 0.006 | -0.05 | (-0.72, 0.63) | 0.894 |
| 24 | 1.14 | (0.26, 2.02) | 0.011 | -0.11 | (-0.85, 0.63) | 0.772 |
| **2% cutoff** | | | | | | |
| 0 | 0.23 | (0.07, 0.40) | 0.006 | 0.19 | (0.03, 0.35) | 0.018 |
| 2 | 0.45 | (0.29, 0.61) | <0.001 | 0.33 | (0.18, 0.47) | <0.001 |
| 4 | 0.48 | (0.33, 0.64) | <0.001 | 0.27 | (0.12, 0.42) | <0.001 |
| 6 | 0.51 | (0.34, 0.68) | <0.001 | 0.22 | (0.05, 0.38) | 0.008 |
| 8 | 0.54 | (0.34, 0.75) | <0.001 | 0.16 | (-0.03, 0.35) | 0.092 |
| 10 | 0.57 | (0.33, 0.82) | <0.001 | 0.11 | (-0.12, 0.33) | 0.348 |
| 12 | 0.61 | (0.32, 0.89) | <0.001 | 0.05 | (-0.21, 0.31) | 0.701 |
| 14 | 0.64 | (0.30, 0.97) | <0.001 | -0.00 | (-0.31, 0.30) | 0.980 |
| 16 | 0.67 | (0.29, 1.05) | <0.001 | -0.06 | (-0.40, 0.29) | 0.739 |
| 18 | 0.70 | (0.27, 1.13) | 0.002 | -0.11 | (-0.50, 0.28) | 0.569 |
| 20 | 0.73 | (0.25, 1.21) | 0.003 | -0.17 | (-0.61, 0.27) | 0.450 |
| 22 | 0.76 | (0.22, 1.30) | 0.005 | -0.22 | (-0.71, 0.26) | 0.366 |
| 24 | 0.79 | (0.20, 1.38) | 0.008 | -0.28 | (-0.81, 0.25) | 0.304 |

Abbreviations: BMI, body mass index; CI, confidence interval; IADL, instrumental activities of daily living; SHARE, *Survey of Health, Ageing and Retirement in Europe*.

Differences in the number of IADL limitations by BMI changes were estimated using segmented linear mixed-effects regression models adjusted for age, sex, marital status, education level, alcohol consumption, current smoking, moderate-to-vigorous physical activity, the number of comorbidities, and body mass index (baseline covariates).

Missing data were imputed using multiple imputation by chained equation, and 100 datasets were imputed. The number of imputed datasets corresponded to approximately twice the highest proportion of participants with at least one missing data, which was found in the SHARE dataset (i.e., 50.4%).

**Supplementary Table 12.** Associations between change in BMI and trajectories of IADL limitations starting two years after stroke (linear mixed-effects regression models)

| **Number of IADL limitations** | | | | | | |
| --- | --- | --- | --- | --- | --- | --- |
| **Changes in BMI** | **Difference at two years** | | | **Difference in changes per year after 2 years post-stroke** | | |
|  | **Estimate** | **95% CI** | **P-value** | **Estimate** | **95% CI** | **P-value** |
| **5% cutoff** | | | | | | |
| Decreased | 0.80 | (0.54, 1.05) | <0.001 | 0.02 | (-0.03, 0.07) | 0.676 |
| Stable | Reference | | | Reference | | |
| Increased | 0.62 | (0.38, 0.86) | <0.001 | -0.01 | (-0.06, 0.03) | 0.676 |
| **2% cutoff** | | | | | | |
| Decreased | 0.44 | (0.21, 0.67) | <0.001 | 0.01 | (-0.03, 0.04) | 0.700 |
| Stable | Reference | | | Reference | | |
| Increased | 0.38 | (0.15, 0.60) | <0.001 | -0.02 | (-0.05, 0.02) | 0.458 |

Abbreviations: BMI, body mass index; CI, confidence interval; IADL, instrumental activities of daily living.

Associations between change in BMI and trajectories of IADL limitations were estimated using segmented linear mixed-effects regression models adjusted for age, sex, marital status, education level, alcohol consumption, current smoking, moderate-to-vigorous physical activity, the number of comorbidities, body mass index, and the number of IADL limitations (baseline covariates).

The follow-up started two years after the wave at which stroke was declared. No breakpoint was used for this model.

**Supplementary Table 13.** Associations between change in BMI and trajectories of IADL limitations after stroke (segmented linear mixed-effects regression models based on self-reported measures of BMI only)

|  | **Number of IADL limitations** | | | | | | | | |
| --- | --- | --- | --- | --- | --- | --- | --- | --- | --- |
| **Changes in BMI** | **Difference at baseline** | | | **Difference in changes per year during 0-2 years after stroke** | | | **Difference in changes per year after 2 years post-stroke** | | |
|  | **Estimate** | **95% CI** | **P-value** | **Estimate** | **95% CI** | **P-value** | **Estimate** | **95% CI** | **P-value** |
| **5% cutoff** | | | | | | | | | |
| Decreased | 0.63 | (0.35, 0.91) | <0.001 | 0.15 | (0.03, 0.27) | 0.006 | -0.01 | (-0.05, 0.06) | 1.000 |
| Stable | Reference | | | Reference | | | Reference | | |
| Increased | 0.57 | (0.31, 0.83) | <0.001 | 0.04 | (-0.07, 0.15) | 0.386 | -0.02 | (-0.07, 0.03) | 0.914 |
| **2% cutoff** | | | | | | | | | |
| Decreased | 0.25 | (0.04, 0.45) | 0.012 | 0.09 | (-0.00, 0.17) | 0.055 | 0.01 | (-0.05, 0.03) | 0.597 |
| Stable | Reference | | | Reference | | | Reference | | |
| Increased | 0.21 | (0.01, 0.40) | 0.023 | 0.08 | (-0.00, 0.16) | 0.055 | -0.01 | (-0.05, 0.02) | 0.498 |

Abbreviations: BMI, body mass index; CI, confidence interval; HRS, *Health and Retirement Study*; IADL, instrumental activities of daily living; SHARE, *Survey of Health, Ageing and Retirement in Europe*.

Associations between change in BMI and trajectories of IADL limitations were estimated using segmented linear mixed-effects regression models adjusted for age, sex, marital status, education level, alcohol consumption, current smoking, moderate-to-vigorous physical activity, the number of comorbidities, and body mass index (baseline covariates).

The breakpoint was set at two years.

This sensitivity analysis was based on HRS and SHARE data that corresponded to self-reported measures of BMI.

**Supplementary Table 14.** Associations between change in BMI measured exactly at two years and trajectories of IADL limitations after stroke (segmented linear mixed-effects regression models)

| **Changes in BMI** | **Number of IADL limitations** | | | | | | | | |
| --- | --- | --- | --- | --- | --- | --- | --- | --- | --- |
|  | **Difference at baseline** | | | **Difference in changes per year during 0-2 years after stroke** | | | **Difference in changes per year after 2 years post-stroke** | | |
|  | **Estimate** | **95% CI** | **P-value** | **Estimate** | **95% CI** | **P-value** | **Estimate** | **95% CI** | **P-value** |
| **5% cutoff** | | | | | | | | | |
| Decreased | 0.62 | (0.33, 0.91) | <0.001 | 0.15 | (0.04, 0.27) | 0.005 | -0.01 | (-0.06, 0.05) | 1.000 |
| Stable | Reference | | | Reference | | | Reference | | |
| Increased | 0.56 | (0.29, 0.83) | <0.001 | 0.05 | (-0.07, 0.16) | 0.319 | -0.02 | (-0.07, 0.03) | 1.000 |
| **2% cutoff** | | | | | | | | | |
| Decreased | 0.23 | (0.02, 0.45) | 0.030 | 0.08 | (-0.01, 0.17) | 0.048 | 0.01 | (-0.03, 0.04) | 0.599 |
| Stable | Reference | | | Reference | | | Reference | | |
| Increased | 0.18 | (-0.03, 0.39) | 0.071 | 0.09 | (0.00, 0.17) | 0.044 | -0.02 | (-0.05, 0.02) | 0.526 |

Abbreviations: BMI, body mass index; CI, confidence interval; IADL, instrumental activities of daily living.

Associations between change in BMI and trajectories of IADL limitations were estimated using segmented linear mixed-effects regression models adjusted for age, sex, marital status, education level, alcohol consumption, current smoking, moderate-to-vigorous physical activity, the number of comorbidities, and body mass index (baseline covariates).

The breakpoint was set at two years.

In these analyses, BMI change was measured at two years exactly, and measures obtained at one, three, and four years were excluded.

**Supplementary Table 15.** Associations between change in BMI and trajectories of IADL limitations after stroke (segmented linear mixed-effects regression models including all interactions between time and the covariates)

| **Changes in BMI** | **Number of IADL limitations** | | | | | | | | |
| --- | --- | --- | --- | --- | --- | --- | --- | --- | --- |
|  | **Difference at baseline** | | | **Difference in changes per year during 0-2 years after stroke** | | | **Difference in changes per year after 2 years post-stroke** | | |
|  | **Estimate** | **95% CI** | **P-value** | **Estimate** | **95% CI** | **P-value** | **Estimate** | **95% CI** | **P-value** |
| **5% cutoff** | | | | | | | | | |
| Decreased | 0.65 | (0.37, 0.93) | <0.001 | 0.16 | (0.05, 0.28) | 0.003 | -0.01 | (-0.07, 0.04) | 1.000 |
| Stable | Reference | | | Reference | | | Reference | | |
| Increased | 0.60 | (0.34, 0.86) | <0.001 | 0.03 | (-0.08, 0.15) | 0.536 | -0.02 | (-0.06, 0.03) | 1.000 |
| **2% cutoff** | | | | | | | | | |
| Decreased | 0.26 | (0.05, 0.46) | 0.007 | 0.09 | (-0.00, 0.18) | 0.037 | 0.00 | (-0.03, 0.04) | 0.794 |
| Stable | Reference | | | Reference | | | Reference | | |
| Increased | 0.22 | (0.02, 0.41) | 0.015 | 0.08 | (0.00, 0.16) | 0.042 | -0.02 | (-0.05, 0.02) | 0.794 |

Abbreviations: BMI, body mass index; CI, confidence interval; IADL, instrumental activities of daily living.

Associations between change in BMI and trajectories of IADL limitations were estimated using segmented linear mixed-effects regression models adjusted for age, sex, marital status, education level, alcohol consumption, current smoking, moderate-to-vigorous physical activity, the number of comorbidities, and body mass index (baseline covariates).

The breakpoint was set at two years.

These analyses included all interactions between time, which was linear, and the covariates.
